# The complete chloroplast genome of *Lilium brownii* F.E.Brown var. *viridulum* Baker (Liliaceae)

**DOI:** 10.1080/23802359.2020.1720534

**Published:** 2020-02-03

**Authors:** Jian Jin, Rongrong Zhou, Hao Liu, Can Zhong, Jing Xie, You Qin, Shuihan Zhang, Yuhui Qin

**Affiliations:** a Institute of Chinese Materia Medica, Hunan Academy of Chinese Medicine, Changsha, P. R. China;; b Graduate School,Hunan University of Chinese Medicine, Changsha, P. R. China;; c College of Pharmacy, Changchun University of Chinese Medicine, Changchun, P. R. China;; d National Resource Center for Chinese Meteria Medica, Chinese Academy of Chinese Medical Sciences, Beijing, China

**Keywords:** Liliaceae, high-throughput sequencing, chloroplast, genome sequence

## Abstract

*Lilium brownii* F.E.Brown var. *viridulum* Baker is a medicinal and food plant that is widely distributed in northern and eastern Asia. Here, we report on the complete chloroplast genome sequence of *L. brownii* var. *viridulum*. The chloroplast genome is 152,665 bp in size and includes two inverted repeat regions of 53,052 bp, which is separated by a large single-copy region of 82,085 bp and a small single-copy region of 17,528 bp. A total of 131 genes were predicted, including 38 tRNA, 8 rRNA, and 85 protein-coding genes. Phylogenetic analysis placed *L. brownii* var. *viridulum* under the family Liliaceae.

*Lilium brownii* F.E.Brown var. *viridulum* Baker, belonging to the family Liliaceae, is widely distributed in northern and eastern Asia (Du et al. 2017). Its bulbs, commonly known as ‘Bai-he’, have long been used as traditional Chinese medicines for treatment of chronic gastritis, pertussis, pneumonia, bronchitis and cough (Ma et al. 2017). ‘Bai-he’ is also regularly consumed as functional food and used to make food supplements, Chinese cuisines, as well as healthy snacks and desserts in China.

Although the chemical constituents, the immune-enhancing activity (Hou et al. 2016) and the complete chloroplast genomes (Hwang et al. 2016; Lee et al. 2016; Song et al. 2016; Su et al. 2019) of some species of Lilium were studied, available genetic resource currently for *Lilium brownii* var. *viridulum* is still scarce. Therefore, it is necessary to develop the genetic resources to protect and use the germplasm of this species. Here, we generated the complete chloroplast genome of *Lilium brownii* var. *viridulum* for further research.

Total genomic DNA was extracted from fresh leaves of *Lilium brownii* var. *viridulum* planted in Botanical Garden, Institute of Chinese Materia Medica, Hunan Academy of Chinese Medicine (N28°13′28.15″, E112°56′26.96″). Additional specimens were kept in Hunan Herbarium of Chinese Traditional Medicine under the collection number HUTM100001.

A genomic library consisting of an insert size of 350 bp was constructed using TruSeq DNA Sample Prep Kit (Illumina, USA) and sequencing was carried out on an Illumina NovaSeq platform. The output was a 6 Gb raw data of 150 bp paired-end reads, further trimmed and assembled using SPAdes (Bankevich et al. 2012). Annotations of chloroplast genome were conducted by the software Geneious (Kearse et al. 2012) and further manually checked by comparison against the *Lilium brownii* complete chloroplast genome (GenBank accession number: KY748296).

The complete chloroplast genome of *Lilium brownii* var. *viridulum* (GenBank accession number: MN906759) is 152,665 bp in length, displaying a quadripartite structure that contains a pair of inverted repeats (IR) regions (53,052 bp), separated by a large single-copy (LSC) region (82,085 bp) and a small single-copy (SSC) region (17,528 bp). There are 131 genes reported, including 85 protein-coding genes, 8 ribosomal RNA genes, and 38 transfer RNA genes. The overall GC content of the chloroplast genome was 37.02%.

For phylogenetic analysis, a maximum-likelihood tree was constructed with 1000 bootstrap replicates using FastTree software (Liu et al. 2011). A subset of 30 species from the family Liliaceae including 24 species from the genus *Lilium* was included, with species *Polygonatum cyrtonema* from Asparagaceae as outgroup. The maximum-likelihood analysis showed that *Lilium brownii* var. *viridulum* is placed under the family Liliaceae, clustered together with other *Lilium* species (Figure 1). The taxonomic status of *Lilium brownii* var. *viridulum* exhibits a closest relationship with *Lilium brownii*. This finding could serve as valuable genomic resources providing insight into conservation and exploitation efforts for this medicinal species.

**Figure 1.**
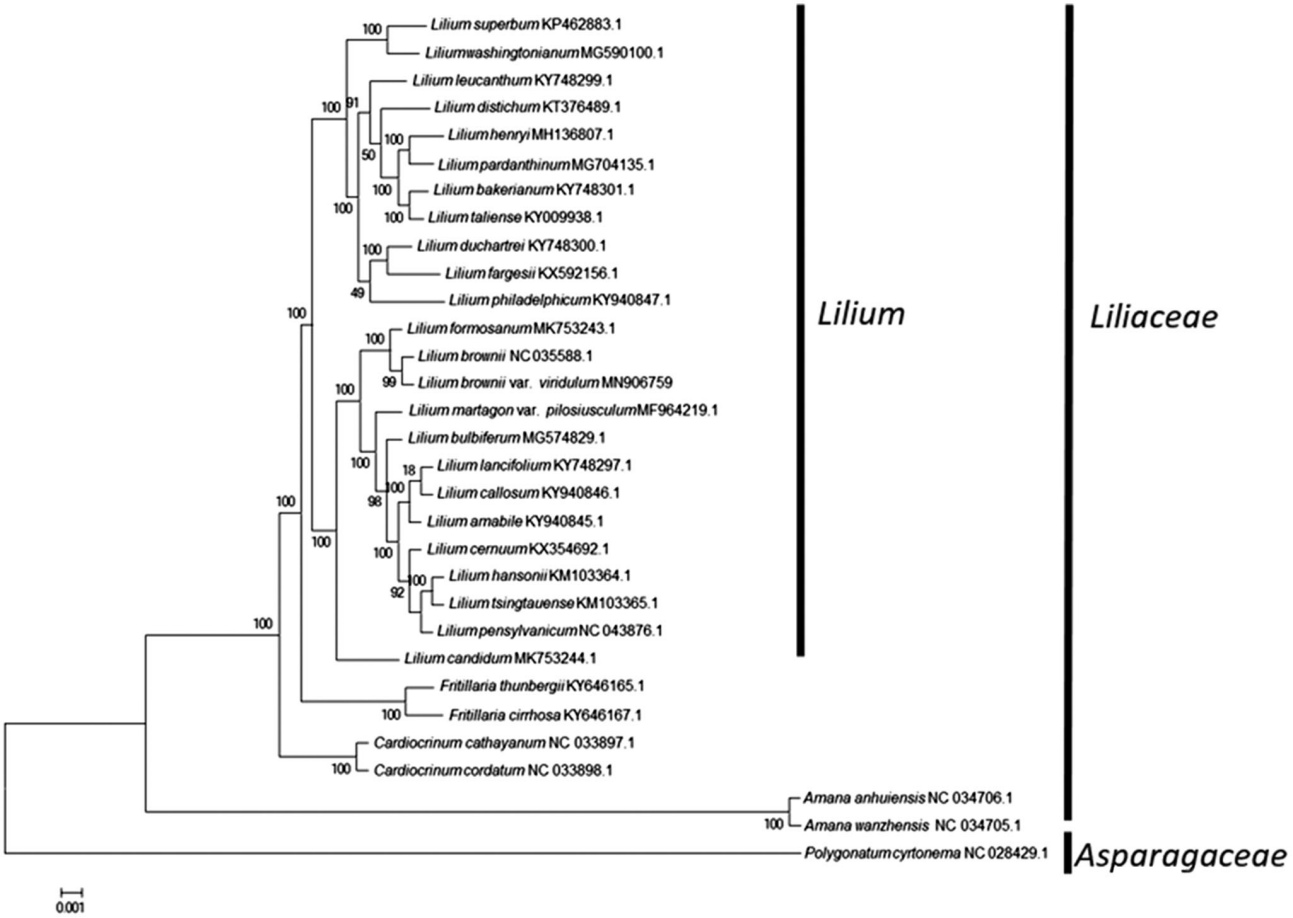
Maximum-likelihood tree based on the complete chloroplast genome sequences of 30 species from the family Liliaceae with *polygonatum cyrtonema* as outgroup. The bootstrap values were based on 1000 replicates.
